# The effect of seed traits on geographic variation in body size and sexual size dimorphism of the seed‐feeding beetle *Acanthoscelides macrophthalmus*

**DOI:** 10.1002/ece3.2364

**Published:** 2016-09-07

**Authors:** Eloísa B. Haga, Marcelo N. Rossi

**Affiliations:** ^1^ Department of Biological Sciences Laboratório de Ecologia Populacional (LEPOP) Federal University of São Paulo (Unifesp) Diadema São Paulo 09941‐510 Brazil

**Keywords:** Ecological variables, evolutionary ecology, intraspecific variation, latitudinal and altitudinal clines, seed quality

## Abstract

Explaining large‐scale patterns of variation in body size has been considered a central question in ecology and evolutionary biology because several life‐history traits are directly linked to body size. For ectothermic organisms, little is known about what processes influence geographic variation in body size. Changes in body size and sexual size dimorphism (SSD) have been associated with environmental variables, particularly for Bruchinae insects, which feed exclusively on seeds during the larval stage. However, the effect of important seed traits on body size variation has rarely been investigated, and whether SSD varies substantially among populations within bruchine species is poorly known. Using the seed‐feeding beetle *Acanthoscelides macrophthalmus* infesting its host plant *Leucaena leucocephala*, we investigated whether specific seed traits (hardness, size, water content, carbon/nitrogen ratio, and phenolic content) were determinant in generating geographic variation in body size and SSD of *A. macrophthalmus*. We also examined the relationships between body size and SSD with latitude and altitude. The body size of both sexes combined was not related to latitude, altitude, and any of the physical and chemical seed traits. However, the female body size tended to vary more in size than the males, generating significant variation in SSD in relation to latitude and altitude. The females were the larger sex at higher latitudes and at lower altitudes, precisely where seed water content was greater. Therefore, our results suggest that water content was the most important seed trait, most severely affecting the females, promoting geographic variation in SSD of *A. macrophthalmus*.

## Introduction

Body size is widely considered one of the most important traits of organisms as it directly affects their physiology (for example, their metabolic rate) and their fitness (Brown et al. 2004; Fairbairn et al. 2007). Variation in body size mostly represents changes in fundamental life‐history traits, such as fecundity, survival, and reproductive success, which have important implications for the adaptation and the evolution of organisms (Reeve et al. 2000; Blanckenhorn and Demont 2004). As a result, biologists have systematically tried to explain patterns in body size variation, particularly at large spatial scales. Previous studies (Bergmann 1847; Blackburn et al. 1999; Blanckenhorn et al. 2006) have shown the presence of latitudinal clines in body size due to the fact that larger organisms in colder climates (i.e., at higher latitudes) may exhibit smaller surface‐to‐volume ratios, thus increasing their capabilities for heat conservation.

Although consistent patterns of body size variation across latitudinal clines have been recorded for endothermic animals and across species (Blackburn and Hawkins 2004), in some cases, temperature does not appear to be the main source of selection (Ashton et al. 2000). This is particularly true for ectothermic animals (Cushman et al. 1993; Ashton and Feldman 2003), which have a greater ability to acclimatize to the surrounding environment (Stevenson 1985; Blanckenhorn et al. 2006). Likewise, negative relationships between body size and latitude have been found for some ectothermic animals (Mousseau 1997; Ashton 2004; Blanckenhorn and Demont 2004). The duration of the growing season, which is short at higher latitudes, appears to be an important factor, in that it limits the foraging time and the development of organisms, as well as their reproductive period (Blanckenhorn and Demont 2004; Blanckenhorn et al. 2006). In addition, it has also been proposed that larger organisms would be selected in drier environments, intensifying their ability to conserve water and to resist dehydration (Wigginton and Dobson 1999; Yom‐Tov and Geffen 2006). Because temperature and humidity are directly affected by altitude, geographic clines in body size can also be associated with altitudinal variation (Karan et al. 2000; Çağlar et al. 2014). Therefore, several environmental variables may exert selection pressures on organisms, thus creating geographic variation in body size.

Within an intraspecific context, resource quality is an important factor driving body size patterns among populations, especially for organisms with short generation times, such as many insect species (Teder and Tammaru 2005). For instance, local adaptations may occur in the long run when populations are exploiting different resources throughout space. In addition, changes in resource quality can markedly affect insect body sizes due to phenotypic plasticity (Amarillo‐Suárez and Fox 2006; Hirst et al. 2015). Hence, resource quality may considerably influence the evolution of body size and other life‐history traits of insects. For insects of the Bruchinae subfamily (Coleoptera: Chrysomelidae: Bruchinae), which feed exclusively on seeds during the larval stage (Ribeiro‐Costa and Almeida 2012), resource quality and quantity are important sources of phenotypic variation among populations (Takakura 2004; Amarillo‐Suárez and Fox 2006). In the case of species where the entire larval feeding stage takes place within a single seed (Silva et al. 2007; Stillwell and Fox 2007; Rossi et al. 2011), resource quality is even more important (Fox et al. 1997; Kestring et al. 2009; Menezes et al. 2010).

Substantial changes in life‐history traits have been shown for some Bruchinae beetles (bruchines hereafter), depending on resource quality. For example, when seeds of different quality are offered to *Stator limbatus*, females oviposit fewer but larger eggs on poor rather than on good quality resources, exhibiting a clear trade‐off between egg size and number (Fox 2000; Savalli and Fox 2002; Czesak and Fox 2003). Although this egg size plasticity may interfere with larval and adult survival, seed quality can also affect larval development, probably determining adult body sizes (Amarillo‐Suárez et al. 2011). However, males and females may respond differently to resource quality during development due to variation in nutritional requirements between the sexes, affecting the magnitude of sexual size dimorphism (SSD). Most taxa exhibit certain levels of SSD (i.e., significant differences in male and female body sizes) (Nylin and Wedell 1994; Fairbairn 2005), but the mechanisms, the sources of selection on males and females, and the environmental variables that generate variation in SSD require a better understanding.

Environmental variables such as temperature, moisture, and the rearing host plant can affect the magnitude of SSD of some bruchines due to local long‐term adaptation, but also because males and females exhibit differences in body size plasticity (Stillwell and Fox 2007, 2009; Stillwell et al. 2007b). This information is very important, because it shows that SSD varies substantially among populations within bruchine species (but considering more than one host plant species); it also confirms that these beetles provide adequate model systems for carrying out experimental studies and for investigating geographic variation in body size and SSD.

Although environmental and climatological variables contribute to explaining body size variation in some bruchines, the effects of relevant seed traits, such as size (Fox et al. 2007; Stillwell et al. 2007a), hardness (Takakura 2004), and water content, have rarely been taken into account, especially for interacting systems composed of a single host plant infested by a single bruchine species. Even for a single host plant, variation in specific seed traits among populations is likely, but the relative influence of such traits on body size and SSD of bruchines is poorly known. In this study, we first proposed that interpopulational variation in seed traits of *Leucaena leucocephala* plants might account for geographic variation in body size of its seed‐feeding beetle *Acanthoscelides macrophthalmus*. *Leucaena leucocephala* shows a wide geographic distribution and *A. macrophthalmus* is very host specific, attributes that make this system an adequate model to investigate our assumptions. Therefore, we also proposed that particular seed traits could be associated with latitudinal and altitudinal trends, explaining possible significant relationships between body size and latitude and altitude. By collecting beetles from 24 populations distributed over 9° of latitude and 1000 m of altitude (approximately), we examined the effect of specific seed traits on interpopulational variation of *A. macrophthalmus* body size and its relationship with latitudinal and altitudinal trends. Also, considering that differences in growth rate and development time in insects are usually found between the sexes (Blanckenhorn et al. 2007; Esperk et al. 2007), particularly when individuals feed on resources of different quality (Davidowitz et al. 2004), we investigated whether seed traits influence the geographic variation in SSD of this seed beetle.

## Methods

### Study system

*Leucaena leucocephala* (Lam.) de Wit (Fabaceae: Mimosoideae) is a tree/bush that can be used for reforestation, firewood, forage for livestock, fertilizer, coal, cellulose production, erosion control, and as a fuel (Elharith et al. 1980; Chagas 1981). While being a multipurpose plant, *L. leucocephala* is also known as a “conflict plant” due to its allelopathic potential in displacing other plants nearby (Neser 1994; Rosa et al. 2007; Williams and Hoagland 2007; Tuda et al. 2009). *Leucaena leucocephala* individuals produce large quantities of dehiscent fruits (about 20 seeds per fruit) and have two to four fructification cycles per year (Smith 1985; Raghu et al. 2005; Tuda et al. 2009). However, the number of fruits and seeds, as well as the fruit and seed sizes, varies considerably among populations. *Leucaena leucocephala* inhabits disturbed areas, being considered a very significant weed in some cases (Lowe et al. 2000). Although this plant is native to Mexico and Central America, due to its invasive properties, it is widely distributed around the globe, occurring in approximately 120 countries (Lowe et al. 2000).

*Acanthoscelides macrophthalmus* (Schaeffer) is a Neotropical bruchine that attacks *L. leucocephala* seeds in most places where this plant occurs (Tuda et al. 2009; Effowe et al. 2010; Shoba and Olckers 2010). *A. macrophthalmus* is, for the most part, host specific to *Leucaena* species (Tuda et al. 2009). It has been suggested that *A. macrophthalmus* is of great importance for *L. leucocephala* control in some countries, because this beetle may limit seed dispersion into new areas, thus reducing the plant's invasion rate (Raghu et al. 2005). Acting as both pre‐ and postdisperser seed predator, this bruchine lays its eggs on *L. leucocephala* pods as well as directly over the seeds (i.e., seeds within dehiscent fruits or after seed dispersion). The larvae have four instars (Wu et al. 2012), and after the emergence, they drill the seed and consume the endosperm and, in most cases, the embryo (Effowe et al. 2010). When reared only with *L. leucocephala* seeds, the average generation time of *A. macrophthalmus* was 34.59 days. Adult females laid approximately 43 eggs and lived from one to two weeks (Effowe et al. 2010).

### Collection of *Acanthoscelides macrophthalmus*

*Leucaena leucocephala* plants are usually found on the roadsides, where they are locally clumped, forming dense populations, although some plants are isolated. Because *L. leucocephala* has a wide geographic distribution in Brazil, *A. macrophthalmus* individuals were collected from 27 populations distributed from the state of Minas Gerais (northernmost) to the state of Rio Grande do Sul (southernmost), near the cities of Belo Horizonte (19° 54′ 31″S; 44° 1′ 34″W) and Porto Alegre (30° 1′ 14″S; 51° 12′ 2″W). Specifically, fruits were collected from populations located on the edge of the Fernão Dias (BR‐381), Régis Bittencourt (BR‐116), and Governador Mário Covas (BR‐101) highways, following a north–south route. Using a GPS (GPSMAP 76CSx – Garmin Olathe, KS), each population had its location (geographic coordinates) and altitude recorded during fruit collection. Populations were distributed over a latitudinal range of 11°, and only six paired populations were <25 km away from each other in a straight line (minimum paired distances: 5.49, 11.97, 14.35, 20.27, 21.95, and 24.41 km). The number of plants that hosted insect populations varied from one plant (five populations) to 10 plants (two populations). On average, each bruchine population was hosted by four *L. leucocephala* trees (±2.6 SD), and fruit collections occurred on the following dates: (1) July 08–14, 2013 and August 08–11 2013 and (2) May 20–26, 2014.

**Figure 1:**
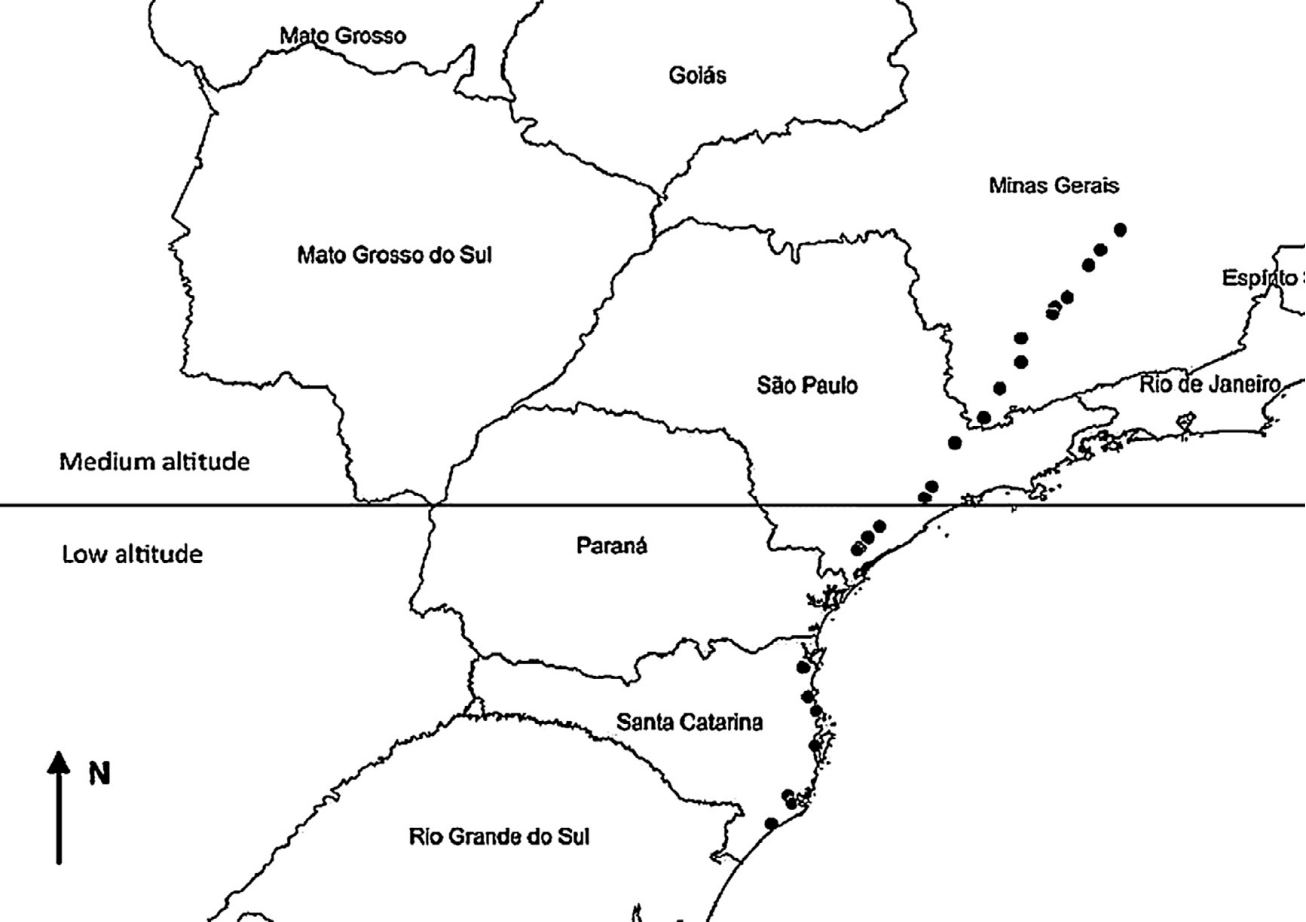
Localities of the 24 populations (each dot = one population) from which fruits and *Acanthoscelides macrophthalmus* individuals were collected. The transversal line indicates the parallel of latitude 24°S, which is the limit that was used to categorize populations in low and medium altitude groups.

For fruit collection, plants were randomly surrounded, and mature fruits located in manually reachable branches were collected. When fruits were available only in high branches, a pruning shears fixed to an aluminum cable extender was used. Fruit collection finished when approximately 100 fruits had been collected per population. Fruits were put in labeled paper bags and transported to the laboratory, where seeds were extracted during fruit dissection. Seeds from plants hosting each bruchine population were homogenized to reduce possible variations (i.e., among trees) and kept in labeled transparent plastic containers (1500 mL) partially covered with a piece of voile fabric (one container per population). The containers were then put into an acclimatized room (27 ± 1°C, 12 h light, 65 ± 5% relative humidity), and the emergence of adult bruchines was monitored over a three‐month period. After the emergence, all bruchines were identified and transferred to flasks (Eppendorf, 1.5 mL) containing ethanol (70%), labeled with the population of origin. Because bruchines did not emerge from the seeds of three populations, data from 24 populations, distributed over a latitudinal range of 9°, were considered in our analysis (Fig. 1).

### Assessment of body size and SSD of *Acanthoscelides macrophthalmus*

Ten males and 10 females of *A. macrophthalmus* from each population were randomly removed from the flasks, carefully dried in ambient air and mounted in triangles fixed with entomological pins. The insects were individually photographed with a digital camera coupled to a stereomicroscope (Leica M205C/DFC450, Wetzlar, Germany) at a standard magnification (2.0×). The following body traits were measured using image analysis software (Leica Image Analysis; version 4.2.0): (1) elytron length; (2) elytron width; and (3) pronotum width. According to Colgoni and Vamosi (2006), the elytron length and pronotum width are the best morphological traits used to estimate body size variation in bruchines. The elytron length was the mean value calculated from the maximum distances along the midlines of both elytra. The elytron width was the mean value calculated from the widest portion of both elytra. The widest portion of pronotum represented its width (Colgoni and Vamosi 2006; Stillwell et al. 2007a). Next, mean values of each morphological trait were computed for each population (Gilchrist et al. 2004; Stillwell et al. 2007a).

Using the Lovich and Gibbons (1992) index, the sexual size dimorphism was computed as follows: SSD = (size of the larger sex/size of the smaller sex) − 1, defined arbitrarily as positive when females are larger than males and negative when males are larger than females. This index, usually known as SDI (SDI = sexual dimorphism index), has the best statistical properties when considering all the other indices that have been suggested (Lovich and Gibbons 1992; Smith 1999; Stillwell et al. 2007a; Stillwell and Fox 2009). Mean values of male and female body sizes (i.e., morphological traits) were used for the SSD calculation for each population.

### Assessment of *Leucaena leucocephala* seed traits

While beetles were measured, intact seeds were removed from the plastic containers and used for the assessment of seed traits. Intact seeds were those without exiting holes made by adult bruchines, penetration holes made by first instar larvae (examined under stereomicroscope), or other damage caused by any number of external factors. Seed hardness was estimated using a needle‐shaped probe (TA09) coupled to a texture analyzer (model CT3‐Brookfield). Seeds were individually placed over a solid rectangular base table embedded right below the probe, and the force (g) required to perforate 0.5 mm of each seed at 1.0 mm/s was recorded. Ten seeds picked at random per population were used for hardness assessment, and each seed represented a replicate. All seeds were discarded after perforation.

For water content estimation, 60 intact seeds from each population were randomly selected and put within three labeled paper bags with 20 seeds each, defining three samples per population (i.e., three replicates). Each sample was first weighed using an analytical scale (model AR2140‐Ohaus) for fresh weight determination. All samples were then oven‐dried (at 105°C for 24 h) and reweighed, and water content determined as follows: water content (%) = ((fresh weight − dry weight) × 100)/fresh weight. After that, the dried seeds were removed from the paper bags and homogenized, forming a single sample per population. Seed dry mass, our proxy for seed size, was estimated by individually weighing 20 seeds taken at random from each population sample. Mean values of seed hardness, seed mass, and water content were calculated for each population.

The dried seeds were milled to a fine powder, and 200 mg per population was used for chemical analyses. Carbon and nitrogen contents were determined from 100 mg of the milled material, and the content of total phenolics was extracted from the remaining 100 mg. Carbon and nitrogen contents were determined using a CHN elemental analyzer (Perkin Elmer 2400‐series II), located in the Chemical Institute (Analytical Center) of the University of São Paulo (USP). To provide a more qualitative investigation and to reduce the number of explanatory variables, the carbon contents were divided by the nitrogen contents and the C/N ratio was calculated for each population and used in the statistical analyses. Total phenolics were quantified by spectrophotometry following the Folin–Ciocalteau method (Singleton et al. 1999; FAO/IAEA 2000), in which gallic acid was used for determining the calibration curve; these analyses were carried out in the Bioorganic Chemical Laboratory “Otto Richard Gottliet” (LABIORG) of the Federal University of São Paulo (Unifesp‐Diadema). Three replicates per population were used for chemical analyses, and for each population, the mean values were calculated for the C/N ratio and total phenolics.

### Data analysis

To create a single variable for body size, we first ran a principal component analysis (PCA) (Lepš and Šmilauer 2003) over the mean population values of the three morphological traits measured (elytron length, elytron width, and pronotum width). Because three SDI values were generated per population, one for each morphological trait, another PCA was run to establish a single variable for SSD. The first principal component (PC1) explained the majority of the variation in body size for both males (95%) and females (96%). The same was observed for SSD, in which the PC1 explained the majority of variation (88%) for the three SDI values calculated. The explanatory power of the remaining variation was minimal for body size and SSD; hence, PC1 was used in subsequent analyses, as the dependent variable in relation to the explanatory variables.

Among the explanatory variables, the altitude was defined as a categorical variable, because two distinct population groups were clearly formed. We categorized the two groups as follows: 1) populations at low altitudes (hereafter “low altitude”), located from 11 m to 46 m (*n *=* *11 populations) and 2) populations at medium altitudes (hereafter “medium altitude”), located from 631 m to 1057 m (*n *=* *13 populations). Populations at low and medium altitudes were found at latitudes higher and lower than 24°S, respectively (Fig. 1). Therefore, the categories included discrete “north” and “south” populations (Fig. 1). To determine whether male and female body sizes (PCs) differed between populations located at low and medium altitude, we ran a linear mixed‐effect model (Crawley 2007) with altitude (low and medium) and sex (male and female) as fixed effect variables, and populations as the random variable. Interaction between the fixed effect variables was also tested. Variation in SSD (PCs) between altitudinal categories was investigated by applying the *t*‐test for independent samples (Zar 1999). The explanatory variables, which could be playing a role in affecting body size and SSD variation, were also compared between both categories by *t*‐tests (independent samples). To examine whether individuals were sexually dimorphic within each category, body size (PC) comparisons between males and females were carried out within each category by paired *t*‐tests (Zar 1999). The Fisher's *F*‐test was run to investigate the homoscedasticity assumption, and the Shapiro–Wilk test was applied to examine for normality (Zar 1999); when one of these conditions was not satisfied, the Mann–Whitney nonparametric test was performed (Zar 1999).

In order to assess whether the response variables of body size and SSD (PCs) were related to the continuous explanatory variables of latitude, seed mass, water content, seed hardness, C/N ratio, and total phenolics, multiple linear regression analyses (Lepš and Šmilauer 2003) were carried out considering mean population values for both the response and explanatory variables. Multiple regressions were conducted separately for male and female body sizes, and for SSD. Before running multiple regressions, however, we investigated for collinearity (i.e., correlations between explanatory variables) (Graham 2003). The investigation of collinearity is important, because once detected, these covariables can be dropped out from the statistical model, resulting, in many cases, in significance for the others (Zuur et al. 2010). To detect collinearity, we used the variance inflation factor (VIF): This method shows whether the standard error of each parameter is inflated with $\sqrt{1/(1-R^{2})}$ (*R* ^2^ is the coefficient of determination) when there is correlation among variables (high *R* ^2^ values); in such cases, the *P*‐values become larger, masking possible significant effects (Zuur et al. 2010). The expression 1/(1 − *R* ^2^) is the first one from the variance equation of a given parameter in linear regression (Zuur et al. 2010). Although some thresholds for VIFs have been proposed (e.g., Montgomery and Peck 1992), Zuur et al. (2010) suggested dropping those variables with VIFs >3, which is considered a rigorous threshold (a more detailed description of the VIF method can be found in Zuur et al. (2010), pages 8–9, and in this article as Supporting information, in the section “VIF Calculation”).

Multiple linear regression analyses were conducted by model simplification, choosing, by parsimony, the least complicated models. By this process, we defined which interactions between explanatory variables and which explanatory variables should be specifically kept in the linear models (Burnham and Anderson 2002; Crawley 2007). When there are many continuous explanatory variables, however, the model structure usually becomes highly complex. In these cases, the risk of overparameterization is high, because there are fewer data points than parameter values, which reduces the explanatory power of the model (Crawley 2007). To deal with overparameterization, it has been suggested not to estimate more than *n*/3 parameters simultaneously, where *n *= number of data points; this is a rule of thumb proposed by Crawley (2007). In this study, we restricted our estimations to 24 (populations)/3 = 8 parameters at a time. To avoid an overparameterized model, interactions were fitted between pairs of variables by randomly rearranging the order of the interaction terms (sample without replacement) (Crawley 2007). Following this process, we obtained 8 (parameters) − 6 (main effects) = 2 interaction terms at a time (main effects must always be included in multiple regressions). Therefore, we began by running eight models for each of the three response variables (female size, male size, and SSD): seven models with two two‐way interaction terms, and another model with only one two‐way interaction term, totaling all the 15 possible interaction pairs. After that, the interaction terms with significant or close‐to‐significant (i.e., 0.05 ≤ *P*‐value ≤ 0.1) results were included into a single model, fitted separately (Crawley 2007). The nonsignificant interaction terms were excluded, and models refitted containing only the explanatory variables. The nonsignificant explanatory variables were also sequentially removed (one at a time), starting with those that showed the highest *P*‐values, until we found the most simplified model (Crawley 2007).

After finding the simplest model, the residuals were plotted against the fitted values to investigate homoscedasticity and the “quantile–quantile plot” (normal Q–Q plot = standardized residuals vs. the theoretical quantiles) was elaborated to examine normality (Crawley 2007). All statistical analyses were processed in R version 3.1.2 (R Development Core Team 2014). Although the R system has the step function, a model simplification tool that automatically finds the lower AIC (Akaike's information criterion), we used the “manual” model simplification procedure, because it is typically more rigorous than the step function at removing variables (and interacting terms) with low explanatory power (Crawley 2007).

## Results

Homoscedasticity was ensured for all the response variables (0.719 ≤ *F* ≤ 1.963; 0.294 ≤ *P* ≤ 0.910) and explanatory variables (0.304 ≤ *F* ≤ 1.389; 0.0545 ≤ *P* ≤ 0.611). With respect to normality, all variables showed normal distributions (0.928 ≤ W ≤ 0.969; 0.087 ≤ *P* ≤ 0.635), with the exception of water content (*W *=* *0.851; *P *=* *0.002), which did not exhibit normality, even after data transformation into arcsin√prop (*W *=* *0.886; *P *=* *0.011). The mean values (±standard error (SE)) for seed mass, seed hardness, phenols, and C/N at low altitude, were, respectively, 0.059 g (±0.003), 2247.818 g (±50.287), 53.896 *μ*g/mL (±2.218), and 5.824% (±0.125); at medium altitude, the mean values (±SE) for seed mass, seed hardness, phenols, and C/N, were, respectively, 0.062 g (±0.003), 2279.962 g (±34.074), 51.364 *μ*g/mL (±1.485), and 5.709% (±0.092). No differences were found between low and medium altitudes for these four explanatory variables (seed mass: *t *=* *0.762; *P *=* *0.454; seed hardness: *t *=* *0.543; *P *=* *0.593; phenols: *t *= −0.975; *P *=* *0.340; C/N: *t *= −0.753; *P *=* *0.459 [22 degrees of freedom (df) for all analyses]).

**Figure 2:**
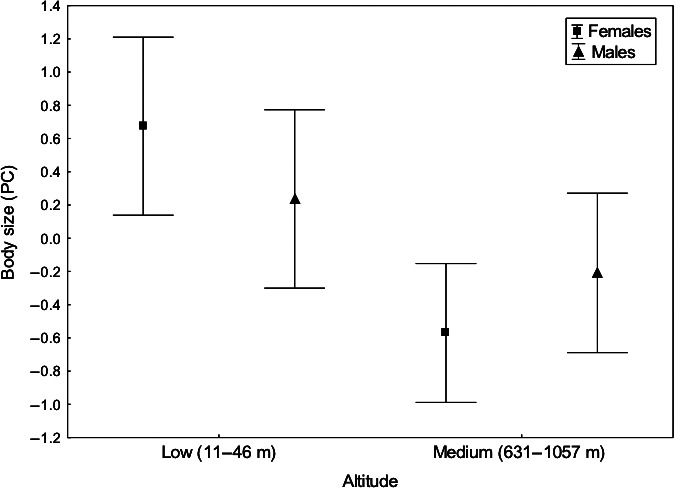
Mean values (±SE) of male and female body sizes (PC1) of *Acanthoscelides macrophthalmus* individuals collected from populations located at low (*N *=* *11) and medium (*N *=* *13) altitudes. Interaction between altitude and sex was significant after running a mixed‐effect model (*P *<* *0.05; see text for details).

**Figure 3:**
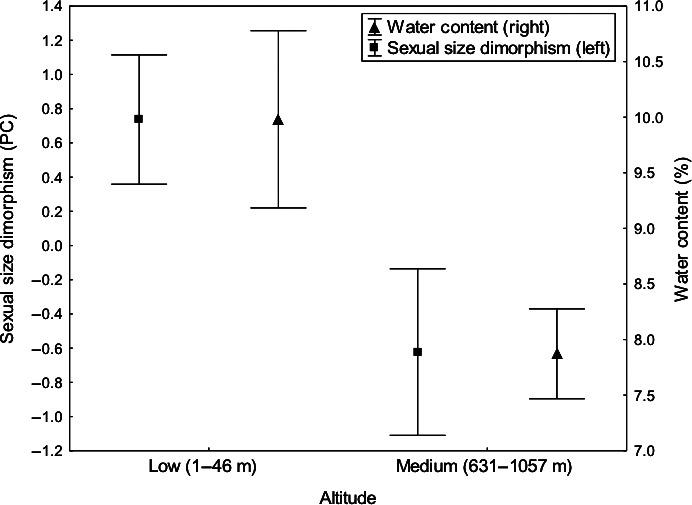
Mean values (±SE) of sexual size dimorphism (PC1) and water content computed from sampling populations (seeds and *Acanthoscelides macrophthalmus* individuals) located at low (*N *=* *11) and medium (*N *=* *13) altitudes. Differences were found between altitude categories for both response variables.

Male and female body sizes did not differ considering the fixed effect variables of altitude (*t *=* *1.792; df = 22; *P *=* *0.087) and sex (*t *=* *1.506; df = 22; *P *=* *0.146) separately. However, a difference was found for the interaction between altitude and sex (*t *= −2.253; df = 22; *P *=* *0.035), and both sexes were, to some extent, larger at lower altitudes (Fig. 2; results for the model intercept: *t *= −1.213; df = 22; *P *=* *0.238). The females were, respectively, larger and smaller than the males at low and medium altitudes. This indicates that the females varied more in size, explaining the significance in the interaction term of the model (Fig. 2). When the body size between males and females was compared within each altitude category, differences were not found (low altitude: *t *= −2.066; df = 10; *P *=* *0.066; medium altitude: *t *=* *1.328; df = 12; *P *=* *0.209). Differences between altitudes were only observed for water content (Mann–Whitney *U*‐test: *U *=* *30; *P *=* *0.018; mean values are presented in Fig. 3) and SSD (*t *= −2.143; df = 22; *P *=* *0.043), with greater values observed at lower altitudes in both cases (Fig. 3). Females were larger than males at low altitudes (positive SSD), but the opposite occurred at medium altitudes (negative SSD) (Fig. 3).

**Figure 4:**
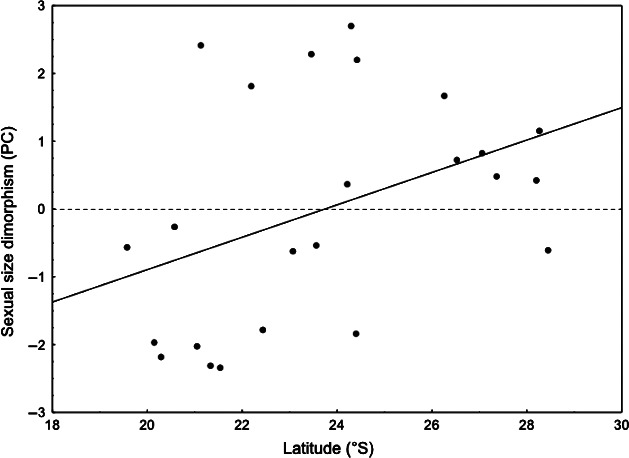
Relationship between sexual size dimorphism (PC1) of *Acanthoscelides macrophthalmus* and latitude (°S). The dashed line indicates the absence of dimorphism. Males and females are the larger sex for values below and above zero (dashed line), respectively. The linear regression line represents the result of the final model after modeling simplification (*P *<* *0.05; see Table 2 for details).

The variance inflation factor (VIF) values for all explanatory variables were below 3.0 (Table 1). Therefore, because collinearity was not detected, all six explanatory variables were included in multiple linear regression analyses. Female body size and SSD individually regressed against latitude, and male body size regressed against latitude, water content, seed mass, and the latitude vs. water content interaction were the minimal adequate models that resulted from the process of simplification (Table 2). Although the body sizes of males and females were not related with any of the explanatory variables, the SSD was positively related with latitude (Table 2; Fig. 4). We found that females were predominantly larger than males with increasing latitude (positive SSD), but the males were the larger sex in most populations located at lower latitudes (negative SSD) (Fig. 4). Results from all interaction terms from the linear modeling process (estimates and the resulting statistics) are presented in Tables S1–S7. The assumptions of homoscedasticity and normality were confirmed (Fig. S1).

**Table 1:** *P*‐values (*t*‐statistic) for the linear multiple regression model and the respective values of the variance inflation factor (VIF) for the full model

Explanatory variables	*P* (full model)^a^	VIF
Latitude	0.327	1.527
Water	0.398	1.536
Hardness	0.824	1.808
Biomass	0.736	2.037
C/N	0.806	1.928
Phenols	0.502	1.348

a In the model, sexual size dimorphism (SSD) was used as the response variable.

**Table 2:** Final linear regression models after the modeling simplification process, considering male and female body sizes and sexual size dimorphism (SSD)

Response variables	Explanatory variables	Estimates	SE	*t*	*P*
Females^a^	Intercept	−5.573	2.861	−1.948	0.0643
	Latitude	0.235	0.119	1.961	0.0626
Males^b^	Intercept	22.012	16.054	1.371	0.186
	Latitude	−0.994	0.654	−1.521	0.145
	Water	−3.545	2.019	−1.758	0.095
	Biomass	47.041	35.767	1.315	0.204
	Latitude × Water	0.141	0.080	1.763	0.094
SSD^c^	Intercept	−5.674	2.743	−2.069	0.0505
	Latitude	0.239	0.1147	2.083	0.0491

a SE (residual) = 1.623; df = 22; *r* ^2^(multiple) = 0.149; *r* ^2^(adjusted) = 0.110; *F *=* *3.846.

b SE (residual) = 1.697; df = 19; *r* ^2^(multiple) = 0.205; *r* ^2^(adjusted) = 0.037; *F *=* *1.223; *P *=* *0.334.

c SE (residual) = 1.556; df = 22; *r* ^2^(multiple) = 0.165; *r* ^2^(adjusted) = 0.127; *F *=* *4.338.

## Discussion

In this study, we showed that the body size of the seed‐feeding beetle *A. macrophthalmus* was not related to altitude (both sexes combined). Although within altitudinal categories males and females did not differ significantly in body size, females were slightly larger and slightly smaller than males at low and medium altitudes, respectively, which explains the significance in body size variation for the parameter of the sex vs. altitude interaction (Fig. 2). This result indicates that females varied more in size than males between altitudes; also, this result explains why the SSD decreased with altitude: SSD was female biased at low altitudes (positive values), but male biased at medium altitudes (negative values). We did not observe latitudinal clines in body size for both sexes. However, we can consider that the result was marginally significant for females (*P *=* *0.063; Table 2), with their body sizes increasing slightly with latitude, which was determinant in creating the latitudinal cline in SSD. Similar clines in dimorphism have occasionally been shown for bruchines. In *S. limbatus*, for example, the females vary more in size with latitude than the males, creating a latitudinal cline in SSD; at lower latitudes, beetles were smaller and more size dimorphic (Amarillo‐Suárez and Fox 2006; Fox et al. 2007; Stillwell et al. 2007a). In the present study, the *A. macrophthalmus* females also varied more in size than the males with latitude. Contrary to altitude, however, the SSD tended from negative to positive values with increasing latitude. This opposite trend occurred because those populations located at lower altitudes were also situated at higher latitudes (south of the equator (°S); see Fig. 1 for altitude and latitude details).

Although geographic variation in SSD within species has been shown for insects, the environmental and/or climatic variables that actually covary with latitude and altitude, generating the clines in SSD, have been explored only scarcely (Blanckenhorn et al. 2006; Stillwell and Fox 2009; Stillwell et al. 2010). Temperature is an abiotic factor, that is, typically associated with changes in the SSD of bruchines, probably due to local adaptations, and because the sexes exhibit different fitness consequences and degrees of plasticity in response to temperature (Stillwell and Fox 2007, 2009). However, ecological and environmental factors other than temperature have been shown to substantially affect the body size and SSD of bruchines. For instance, it has been found that temperature did not explain geographic variation in *S. limbatus* body size, which was actually correlated with seed size, humidity, and seasonality (Stillwell et al. 2007a; Stillwell and Fox 2009); in this case, although the SSD was positively correlated with humidity, the latitudinal cline in SSD was maintained after eliminating humidity as an explanatory variable, which suggests that the investigation of other environmental variables is important in determining the causes of geographic variation in SSD.

Host plant characteristics, expressed by the use of seeds from different host plants, and by the quantification of physical and chemical seed traits (i.e., seed quality/quantity), have also been suggested as important sources of selection on female and male body size in bruchines (Messina 2004; Stotz et al. 2013). Studying whether seed traits influence bruchines’ body size is relevant, considering their inherent biology. Adult bruchines obtain most of their energy during larval feeding within seeds; hence, it is expected that seed quality (including seed size) may account for significant variation in adult body sizes (González‐Teuber et al. 2008; Menezes et al. 2010). It is therefore surprising that there is a lack of studies that examine the degree of variation in bruchines’ body size and SSD in response to seed traits, considering a single host plant and bruchine species. After exploring the effect of several seed traits on geographic variation in body size and SSD of *A. macrophthalmus*, our results strongly suggest that a difference in seed water content was the most powerful explanatory variable, especially for producing SSD clines. By feeding on low water stressed plants (or plant parts), the performance of insect herbivores may increase, because greater water availability may help the digestibility of plant tissues and may facilitate nutrient assimilation (e.g., in the case of sapsuckers) (Huberty and Denno 2004). On the other hand, plant resistance to herbivore insects may increase greatly depending on water content in plant tissues, as water availability influences directly and indirectly numerous plant chemical traits, such as defensive secondary compounds (Huberty and Denno 2004; Bosu and Wagner 2007). Therefore, the life cycle and the development of some insect feeding guilds might be markedly affected by water availability (Huberty and Denno 2004; Mody et al. 2009), influencing their adult body sizes.

It has been argued that bruchines’ bodies contain approximately 50% of water soon after emerging from seeds and that the larvae use metabolic water (Johnson and Kistler 1987; Ribeiro‐Costa and Almeida 2012). These findings support our suggestion that seed water content might be an essential variable in determining body size and SSD variation in bruchines. On the other hand, we are aware that the rearing method we used has some limitations, due to the fact that the developmental stage of larvae within the seeds could not be controlled after fruit collection, which means that some emerging adults may have had most of their larval development at laboratory conditions. In addition, the effect of temperature cannot be disregarded completely. For example, using a database consisting only of arthropods, Horne et al. (2015) found significant correlation between body size and temperature in the field, resulting particularly in changes in body size plasticity, which were associated with voltinism (i.e., season‐length trade‐offs). It is possible that at higher latitudes (i.e., in the south), mean annual temperatures are lower, contributing to the observed cline in SSD of *A. macrophthalmus*. However, as the *L. leucocephala* individuals were located on the roadsides with few weather stations reasonably near, the collection of temperature data was not feasible. It is interesting to note, however, that our study shows a clear connection between the explanatory variables of latitude, altitude, and water content. The SSD of *A. macrophthalmus* decreased at medium altitudes with the concomitant decrease in seed water content. Although SSD was positively related with latitude, after looking carefully at Figure 4, we realize that the latitude of 24°S divides the plot into two distinct clouds of points; most points on the left side represent male‐biased SSD, while most points on the right side show female‐biased SSD. In summary, the plants that had seeds with lower water contents were those located at lower latitudes but also at medium altitudes, suggesting that the latitudinal cline in SSD was caused by marked differences in seed water content between altitudinal classes.

Variation in SSD is likely to occur due to sex differences in growth rate and development time in insects (Blanckenhorn et al. 2007; Esperk et al. 2007), especially when individuals are raised on resources that differ in quality (Davidowitz et al. 2004). This means that males and females differ in body size plasticity, producing intraspecific variation in SSD. In insects, usually the females are more nutritionally sensitive than the males (Telang et al. 2001), being more severely affected by resource quality. For example, Hirst et al. (2015) showed that SSD was not systematically dependent on changes in temperature considering all the 17 arthropod orders studied; they suggested that juvenile density and food quantity/quality promote different adaptive effects on SSD, particularly, greater plasticity in female sizes. Interesting experimental studies have shown that the bruchine *Callosobruchus maculatus* exhibits plasticity in body size according to environmental variables such as the rearing host plant and temperature (or the interaction of both) (Stillwell et al. 2007b). Therefore, it is possible that the geographic variation in SSD observed in *A. macrophthalmus* occurred because the sexes exhibit different responses to seed water content (i.e., phenotypic plasticity), suggesting that *A. macrophthalmus* females vary more in size than males due to their greater sensitivity to water limitation.

Using climatic variables (weather stations data), Stillwell et al. (2007a) found that adults of the bruchine *S. limbatus* were larger at low‐humidity sites. The possible explanation for this pattern is that in drier environments, larger insects could conserve water more efficiently (Hoffmann and Harshman 1999), which is probably associated with the fact that the adults do or do not need access to water to reproduce (Stillwell et al. 2007a). In this study, however, we showed an opposite trend; *A. macrophthalmus* individuals were actually larger on seeds with higher water content, even though body size alone did not differ between populations containing seeds that differed in this trait. Because seed traits directly affect the larval and not the adult stage, our findings suggest that *L. leucocephala* seeds with low water content negatively affect the growth rate and the development time of *A. macrophthalmus* larvae, and probably other life‐history traits not recorded here, such as fecundity and survival. Considering that both the larvae and the adults can have access to water, how these stages differ in water requirements is a crucial question, which certainly will help our understanding of the actual causes of body size and SSD variation in seed‐feeding beetles.

It has been shown that females of some bruchine species lay more eggs when fed with water, proteins, and sugar (Tatar and Carey 1995). Shortages of water and nutrients can also affect the reproductive potential of females later in life, which is related to the rate at which females mate (i.e., nuptial gifts from the ejaculate) (Arnqvist et al. 2005). Furthermore, it has been suggested that adult bruchines frequently experience dehydration because they do not feed (or feed little) and that the females may gain additional water supply by ejaculates (Leroi 1981; Arnqvist et al. 2005). Thus, these findings are indications that the fecundity of bruchine females is significantly affected by water supply. Because fecundity is frequently positively correlated with body size (e.g., Preziosi et al. 1996), we suggest that seed water content may affect the females to a greater extent than the males, explaining the greater variation observed in the body sizes of *A. macrophthalmus* females.

Seed size is another very important seed trait that may alter body size and SSD because changes in this trait represent variation in resource availability to the larvae (i.e., resource quantity) (González‐Teuber et al. 2008; Amarillo‐Suárez et al. 2011). However, seed mass was not associated with either body size or SSD in *A. macrophthalmus*. Most studies that have examined seed size effects on body size have only evaluated differences between different species of host plant, rather than differences in seed size within the same species of plant (Amarillo‐Suárez and Fox 2006; Amarillo‐Suárez et al. 2011). We believe that other important seed traits such as hardness and content of specific chemical compounds should vary more between species than within the same plant species. Whether these traits covary with seed size, revealing their relative effects on body size and SSD, requires a deeper investigation.

The present study has confirmed that interpopulational variation in SSD of *A. macrophthalmus* was affected by changes in specific seed traits, in this case, water content. This geographic variation in SSD was primarily caused by greater variation in female sizes. Our predictions, that particular seed traits could be associated with latitudinal and altitudinal trends, were also confirmed, because seed water content was strictly linked to latitudinal and altitudinal variation. Our findings also have implications for the understanding of body size variation of other taxa. For example, Molleman et al. (2011) studied weight loss in many species of Lepidoptera (pupal and adult live weights) and observed that water loss was the primary variable responsible for much of the pupal weight loss upon adult emergence, affecting the water content of adults; the weight loss differed between the sexes, usually being male biased. Therefore, these results suggest that water content is critical in facilitating metamorphosis. Even for vertebrates, it has been experimentally shown that the egg mass of snakes can be affected by the amount of water taken up by the eggs (Brown and Shine 2005). Differences in the evaporative water loss rate in lizards (i.e., physiological performance) have also been explained by differences in body mass between males and females (Cullum 1998).

Finally, as far as we know, this is the first study to show the importance of seed water content in driving SSD variation in bruchines. Whether variation in SSD among *A. macrophthalmus* populations has a genetic basis is still unknown. Environmental factors (including seed traits) that vary geographically could differently influence the selection on males and females of *A. macrophthalmus*. Therefore, experimental studies are needed, which would unravel more precisely whether the *A. macrophthalmus* populations differ genetically, and whether the sexes differ in fitness consequences according to body size, according to the degree of plasticity, or whether the variation is due to a combination of factors.

## Conflict of Interest

None declared.

## Supporting information


**VIF Calculation.** Description of the protocol used to compute the VIFs.
**Table S1.** Results from eight linear multiple regression analyses for male and female body sizes, considering the interactions between pairs of explanatory variables.
**Table S2.** Results generated from multiple linear regression analyses between male body size (response variable) and all explanatory variables, considering the interaction between latitude and water content. Analysis conducted after detecting a close‐to‐significant result for this interaction.
**Table S3.** Results generated from multiple linear regression analyses between male body size (response variable) and the explanatory variables of latitude, water content, hardness, biomass, C/N ratio, and the interaction between latitude and water content. Analysis conducted after removing the variable of phenolic content.
**Table S4.** Results generated from multiple linear regression analyses between male body size (response variable) and the explanatory variables of latitude, water content, biomass, C/N ratio, and the interaction between latitude and water content. Analysis conducted after removing the variable of seed hardness.
**Table S5.** Results from the eight linear multiple regression analyses for sexual size dimorphism as the response variable, considering the interactions between pairs of explanatory variables.
**Table S6.** Results generated from multiple linear regression analyses between sexual size dimorphism (response variable) and all explanatory variables, taking into account the interaction between biomass and seed hardness. Analysis conducted after detecting a significant result for this interaction.
**Table S7.** Results generated from multiple linear regression analyses between sexual size dimorphism (response variable) and all explanatory variables. Analysis conducted after removing the interaction between biomass and seed hardness.

**Fig. S1.** Plots of the residuals vs. the fitted values (A) and of the standardized residuals vs. the theoretical quantiles (B), showing homoscedasticity and normality trends, respectively.
