# Supplementary material for: The effect of seed traits on geographic variation in body size and sexual size dimorphism of the seed‐feeding beetle Acanthoscelides macrophthalmus
Source: Ecol Evol. 2016 Sep 7;6(19):6892–905. doi: 10.1002/ece3.2364 (PMC5513244; doi:10.1002/ece3.2364)
Supplement: Supplementary file 1 — VIF Calculation. Description of the protocol used to compute the VIFs. Table S1. Results from eight linear multiple regression analyses for male and female body sizes, considering the interactions between pairs of explanatory variables. Table S2. Results generated from multiple linear regression analyses between male body size (response variable) and all explanatory variables, considering the interaction between latitude and water content. Analysis conducted after detecting a close‐to‐significant result for this interaction. Table S3. Results generated from multiple linear regression analyses between male body size (response variable) and the explanatory variables of latitude, water content, hardness, biomass, C/N ratio, and the interaction between latitude and water content. Analysis conducted after removing the variable of phenolic content. Table S4. Results generated from multiple linear regression analyses between male body size (response variable) and the explanatory variables of latitude, water content, biomass, C/N ratio, and the interaction between latitude and water content. Analysis conducted after removing the variable of seed hardness. Table S5. Results from the eight linear multiple regression analyses for sexual size dimorphism as the response variable, considering the interactions between pairs of explanatory variables. Table S6. Results generated from multiple linear regression analyses between sexual size dimorphism (response variable) and all explanatory variables, taking into account the interaction between biomass and seed hardness. Analysis conducted after detecting a significant result for this interaction. Table S7. Results generated from multiple linear regression analyses between sexual size dimorphism (response variable) and all explanatory variables. Analysis conducted after removing the interaction between biomass and seed hardness. [file ECE3-6-6892-s001.docx]

**VIF Calculation**

Variance inflation factors (VIFs) are calculated using the *R*^2^ values from linear regression models in which the response variable of a given regression model is actually an explanatory variable, and the other explanatory variables remain qualitatively unchanged (Zuur et al. 2010). After that, the explanatory variable with the highest VIF is dropped and the process is sequentially repeated, recalculating the VIFs. However, here we calculated the VIFs from a linear regression model containing all the explanatory variables and sexual size dimorphism (SSD) as the response variable. Through this protocol, the *R* statistical software (R Development Core Team 2014) automatically indicates which are the explanatory variables and computes the VIFs as if each explanatory variable had been used, one at a time, as the response variable in regressions. We adopted 3 as our cut-off criterion for the VIFs.

**Table S1.** Results from eight linear multiple regression analyses for male and female body sizes, considering the interactions between pairs of explanatory variables.

| Response variables | Explanatory variables* | Estimates | SE | *t* | *P* |
| --- | --- | --- | --- | --- | --- |
| Females | Hardness*Phenols | -7.681x10^-4^ | 5.865x10^-4^ | -1.310 | 0.210 |
|  | Biomass*C/N | 1.003x10^2^ | 1.155x10^2^ | 0.869 | 0.399 |
|  | Latitude*Biomass | -1.430x10^1^ | 1.919x10^1^ | -0.745 | 0.468 |
|  | Latitude*C/N | -1.908x10^-2^ | 3.956x10^-1^ | -0.482 | 0.636 |
|  | C/N*Phenols | 0.123 | 0.164 | 0.752 | 0.464 |
|  | Latitude*Phenols | -0.017 | 0.028 | -0.588 | 0.565 |
|  | C/N*Hardness | -0.000 | 0.007 | -0.017 | 0.987 |
|  | Latitude*Water | 0.123 | 0.097 | 1.260 | 0.227 |
|  | Water*Phenols | -5.243x10^-4^ | 3.947x10^-2^ | -0.013 | 0.990 |
|  | Water*Biomass | -1.440x10^1^ | 2.864x10^1^ | -0.503 | 0.622 |
|  | Water*Hardness | 1.520x10^-3^ | 1.645x10^-3^ | 0.924 | 0.370 |
|  | Latitude* Hardness | -1.3620x10^-3^ | 8.854x10^-4^ | -1.539 | 0.145 |
|  | Biomass*Phenols | -8.104 | 6.230 | -1.301 | 0.213 |
|  | Biomass* Hardness | 0.077 | 0.340 | 0.227 | 0.824 |
|  | Water*C/N | 0.787 | 0.702 | 1.120 | 0.279 |
| Males | Biomass*Phenols | -8.166 | 7.574 | -1.078 | 0.298 |
|  | Water*Biomass | 3.028 | 29.582 | 0.102 | 0.920 |
|  | Latitude*Biomass | -1.756x10^1^ | 1.715x10^1^ | -1.024 | 0.322 |
|  | Biomass*C/N | 1.040x10^2^ | 1.143x10^2^ | 0.910 | 0.377 |
|  | Latitude*Hardness | -1.158x10^-3^ | 9.844x10^-4^ | -1.177 | 0.258 |
|  | Water*Hardness | 5.604x10^-4^ | 1.829x10^-3^ | 0.306 | 0.764 |
|  | Latitude*Phenols | -0.007 | 0.024 | -0.299 | 0.769 |
|  | Hardness*Biomass | -0.166 | 0.405 | -0.410 | 0.688 |
|  | Hardness*C/N | 3.803x10^-3^ | 8.955x10^-3^ | 0.425 | 0.677 |
|  | Water*C/N | 6.811x10^-1^ | 9.946x10^-1^ | 0.685 | 0.504 |
|  | Latitude*Water | 0.180 | 0.101 | 1.794 | 0.093 |
|  | Latitude*C/N | -0.140 | 0.326 | -0.431 | 0.672 |
|  | Water*Phenols | -0.013 | 0.038 | -0.333 | 0.744 |
|  | C/N*Phenols | 0.039 | 0.130 | -0.297 | 0.770 |
|  | Hardness*Phenols | -6.462x10^-4^ | 5.193x10^-4^ | -1.244 | 0.231 |

*For each response variable: seven models with two two-way interaction terms, and one model with only one two-way interaction term (15 interaction pairs).

**Table S2.** Result from multiple linear regression analysis between male body size (response variable) and all explanatory variables, considering the interaction between latitude and water content. Analysis conducted after detecting a close-to-significant result for this interaction.

| Explanatory variables | Estimates | SE | *t* | *P* |
| --- | --- | --- | --- | --- |
| Intercept | 18.856 | 18.063 | 1.044 | 0.312 |
| Latitude | -1.235 | 0.776 | -1.590 | 0.131 |
| Water | -4.160 | 2.324 | -1.790 | 0.092 |
| Hardness | -0.001 | 0.004 | 0.375 | 0.713 |
| Biomass | 48.358 | 50.656 | 0.955 | 0.354 |
| C/N | 0.830 | 1.477 | 0.562 | 0.582 |
| Phenols | 0.013 | 0.070 | 0.192 | 0.845 |
| Latitude*Water^†^ | 0.167 | 0.093 | 1.792 | 0.092 |

SE (residual) = 1.816; *df* = 16; r^2^(multiple) = 0.233; r^2^(adjusted) = -0.103; *F* = 0.693; *P* = 0.678

^†^*P* = 0.093 in the previous analysis (see table S1).

**Table S3.** Result from multiple linear regression analysis between male body size (response variable) and the explanatory variables latitude, water content, hardness, biomass, C/N relation and considering the interaction between latitude and water content. Analysis conducted after removing the variable phenolic contents.

| Explanatory variables | Estimates | SE | *t* | *P* |
| --- | --- | --- | --- | --- |
| Intercept | 18.856 | 17.421 | 1.059 | 0.305 |
| Latitude | -1.222 | 0.751 | -1.626 | 0.1222 |
| Water | -4.119 | 2.248 | -1.833 | 0.0844 |
| Hardness | 0.001 | 0.004 | 0.378 | 0.710 |
| Biomass | 50.582 | 47.903 | 1.056 | 0.306 |
| C/N | 0.957 | 1.285 | 0.745 | 0.467 |
| Latitude*Water | 0.166 | 0.090 | 1.834 | 0.084 |

SE (residual) = 1.764 *df* = 17; r^2^(multiple) = 0.231; r^2^(adjusted) = -0.041; *F* = 0.85; *P* = 0.550

**Table S4.** Result from multiple linear regression analysis between male body size (response variable) and the explanatory variables latitude, water content, biomass, C/N relation and considering the interaction between latitude and water content. Analysis conducted after removing the variable seed hardness.

| Explanatory variable | Estimates | SE | *t* | *P* |
| --- | --- | --- | --- | --- |
| Intercept | 19.870 | 16.600 | 1.197 | 0.247 |
| Latitude | -1.128 | 0.693 | -1.629 | 0.121 |
| Water | -3.886 | 2.109 | -1.842 | 0.082 |
| Biomass | 59.471 | 40.719 | 1.461 | 0.161 |
| C/N | 0.798 | 1.185 | 0.673 | 0.509 |
| Latitude*Water | 0.155 | 0.084 | 1.849 | 0.081 |

SE (residual) = 1.722; *df* = 18; r^2^(multiple) = 0.224; r^2^(adjusted) = 0.009; *F* =1.041; *P* = 0.424

Hereafter, after removing the variable C/N relation, the most simplified model was obtained (see Table 2 in the manuscript).

**Table S5.** Results from the eight linear multiple regression analyses for sexual size dimorphism as the response variable, considering the interactions between pairs of explanatory variables.

| Explanatory variables | Estimates | SE | *t* | *P* |
| --- | --- | --- | --- | --- |
| Biomass*Hardness | 7.615x10^-1^ | 3.462x10^-1^ | 2.200 | *0.044* |
| Latitude*Hardness | 1.092x10^-3^ | 8.347x10^-4^ | 1.309 | 0.210 |
| Latitude*Phenols | 1.541x10^-2^ | 2.184x10^-2^ | 0.706 | 0.491 |
| Water*Phenols | -4.304x10^-3^ | 3.706x10^-2^ | -0.116 | 0.909 |
| Biomass*Phenols | -1.747x10^0^ | 6.858x10^0^ | -0.255 | 0.802 |
| Latitude*Biomass | 8.502x10^0^ | 1.604x10^1^ | 0.530 | 0.604 |
| Hardness*Phenols | 3.670x10^-4^ | 4.896x10^-4^ | 0.750 | 0.465 |
| Water*Biomass | -8.231x10^0^ | 2.416x10^1^ | -0.341 | 0.738 |
| Latitude*C/N | -0.313 | 0.334 | -0.938 | 0.363 |
| Water*C/N | 0.956 | 0.792 | 1.207 | 0.246 |
| Hardness*C/N | -5.515x10^-4^ | 3.213x10^-2^ | -0.032 | 0.975 |
| Biomass*C/N | -6.209x10^1^ | 8.803x10^2^ | -0.236 | 0.817 |
| Latitude*Water | -4.695x10^-2^ | 1.351x10^-1^ | -0.348 | 0.733 |
| Water*Hardness | 8.849x10^-4^ | 2.219x10^-3^ | 0.399 | 0.696 |
| C/N*Phenols | 1.384x10^-2^ | 1.099x10^-1^ | 0.126 | 0.901 |

*Seven models with two two-way interaction terms, and one model with only one two-way interaction term (15 interaction pairs).

**Table S6.** Result from multiple linear regression analysis between sexual size dimorphism (response variable) and all explanatory variables, considering the interaction between biomass and seed hardness. Analysis conducted after detecting a significant result for this interaction.

| Explanatory variables | Estimates | SE | *t* | *P* |
| --- | --- | --- | --- | --- |
| Intercept | 6.044x10^1^ | 3.746x10^1^ | 1.614 | 0.126 |
| Latitude | 1.217x10^-1^ | 1.463x10^-1^ | 0.832 | 0.418 |
| Water | 2.386x10^-1^ | 1.837x10^-1^ | 1.299 | 0.212 |
| Hardness | -2.906x10^-2^ | 1.659x10^-2^ | -1.751 | 0.099 |
| Biomass | -1.194x10^3^ | 6.792x10^2^ | -1.757 | 0.098 |
| C/N | 1.311x10^-1^ | 1.260x10^0^ | -0.104 | 0.918 |
| Phenols | -5.153x10^-4^ | 6.577x10^-2^ | -0.008 | 0.994 |
| Biomass*Hardness^†^ | 5.212x10^-1^ | 3.00x10^-1^ | 1.737 | 0.102 |

SE (residual) = 1.591; *df* = 16; r^2^(multiple) = 0.365; r^2^(adjusted) = -0.087; *F* = 1.312; *P* = 0.307

^†^*P* = 0.044 in the previous analysis (see table S5).

**Table S7.** Result from multiple linear regression analysis between sexual size dimorphism (response variable) and all explanatory variables. Analysis conducted after removing the interaction between biomass and seed hardness.

| Explanatory variables | Estimates | SE | *t* | *P* |
| --- | --- | --- | --- | --- |
| Intercept | -2,028x10^0^ | 1,111x10^1^ | -0,183 | 0,857 |
| Latitude | 1,547x10^-1^ | 1,534x10^-1^ | 1,008 | 0,327 |
| Water | 1,637x10^-1^ | 1,889x10^-1^ | 0,867 | 0,398 |
| Hardness | -7,512x10^-4^ | 3,316x10^-3^ | -0,227 | 0,824 |
| Biomass | -1,609x10^1^ | 4,692x10^1^ | -0,343 | 0,736 |
| C/N | 3,310x10^-1^ | 1,327x10^0^ | 0,249 | 0,806 |
| Phenols | -4,411x10^-2^ | 6,431x10^-2^ | -0,686 | 0,502 |

EP (residual) = 1.683; *df* = 17; r^2^(multiple) = 0.245; r^2^(adjusted) = -0.022; *F* = 0.918; *P* = 0.506

Hereafter, after removing the variable seed hardness, the most simplified model was obtained after sequentially removing the non-significant explanatory variables with the highest *P*-values (see text explanations and Table 2 in the manuscript).

**Fig. S1.** Plots for the residuals *vs.* the fitted values (A) and for the standardized residuals *vs.* the theoretical quantiles (B), showing homoscedasticity and normality trends, respectively.
