# Supplementary figures and images for: The effect of seed traits on geographic variation in body size and sexual size dimorphism of the seed‐feeding beetle Acanthoscelides macrophthalmus
Source: Ecol Evol. 2016 Sep 7;6(19):6892–905. doi: 10.1002/ece3.2364 (PMC5513244; doi:10.1002/ece3.2364)

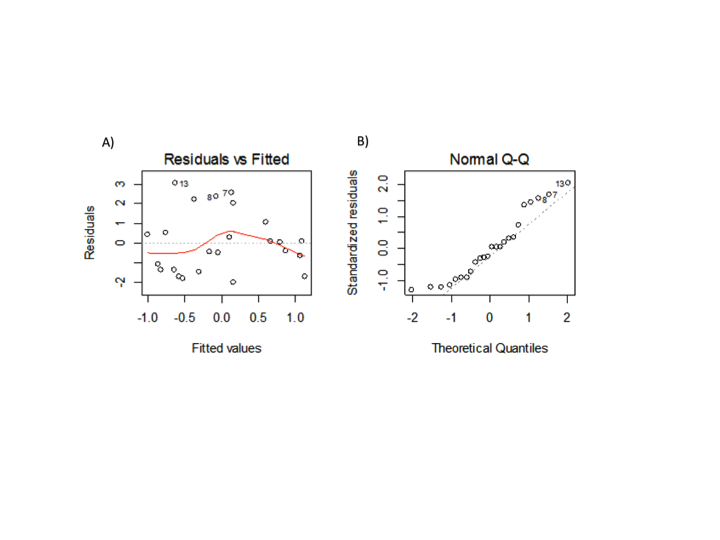

Supplement: Supplementary file 2 — Fig. S1. Plots of the residuals vs. the fitted values (A) and of the standardized residuals vs. the theoretical quantiles (B), showing homoscedasticity and normality trends, respectively. [file ECE3-6-6892-s002.png]
